# On ways to overcome the magical capacity limit of working memory

**DOI:** 10.1371/journal.pbio.2005867

**Published:** 2018-04-19

**Authors:** Zsolt Turi, Ivan Alekseichuk, Walter Paulus

**Affiliations:** 1 Department of Clinical Neurophysiology, University Medical Center Goettingen, Georg-August University of Goettingen, Goettingen, Germany; 2 Department of Biomedical Engineering, University of Minnesota, Minneapolis, Minnesota, United States of America

## Abstract

The ability to simultaneously process and maintain multiple pieces of information is limited. Over the past 50 years, observational methods have provided a large amount of insight regarding the neural mechanisms that underpin the mental capacity that we refer to as “working memory.” More than 20 years ago, a neural coding scheme was proposed for working memory. As a result of technological developments, we can now not only observe but can also influence brain rhythms in humans. Building on these novel developments, we have begun to externally control brain oscillations in order to extend the limits of working memory.

Brain rhythms or neural oscillations are periodic fluctuations of the electric fields in the cerebral cortex. Neurons, temporally synchronized groups of neurons creating neural assemblies, and brain networks operate in a rhythmic and synchronized fashion. Network oscillations, the aggregate oscillatory activity of a large number of neural assemblies, provide the substrate for information processing and facilitate the local-to-global communication crucial for cognition [1]. Working memory depends on the dynamic oscillatory interaction of brain-wide neural networks. Although correlational studies already suggested that the interaction of neural oscillations play an important role in the working memory capacity limit, Wolinski and colleagues [2] tested this proposition by using a noninvasive electrical brain stimulation technique in order to modulate rather than just observe neural oscillations.

Neural oscillations arise at different frequencies, from the ultra-slow (<1 Hz), over delta (1–3 Hz), theta (3–7 Hz), alpha (8–12 Hz), beta (13–25 Hz), to gamma (>25 Hz) frequency bands. Slower and faster rhythms interact by cross-frequency coupling [3]. The best-known type of such coupling is the phase–amplitude cross-frequency coupling, in which the phase of lower frequency oscillations—e.g., the theta rhythm—correlates with the amplitude of higher frequencies—e.g., the gamma rhythm.

Working memory refers to the ability to encode, maintain, and manipulate multiple pieces of information for a short period in a changing environment. The underlying neural mechanisms must satisfy two seemingly opposing criteria. They should concurrently store new sensory inputs while preventing new items from interfering with already existing memory items. It has been hypothesized that theta–gamma phase–amplitude coupling provides the necessary neural coding mechanism for maintaining working memory and preventing interference [4].

A core postulate of the theta–gamma neural code model is that during working memory maintenance, each cycle in the gamma rhythm encodes an individual memory item [4]. The theta–gamma neural coding scheme organizes “item-specific” gamma cycles in such a way that each gamma cycle has a certain “position” at a specific phase of the theta cycle (Fig 1), as shown by electrophysiological recording both from rodent hippocampi and from intracranial electrodes in humans [5,6]. One important assumption of the model is that the frequency of the theta oscillation determines the limit of the working memory’s capacity. Because the gamma subcycles utilize unique phase codes to represent discrete memory items, the lower the theta frequency is, the more gamma subcycles it can host. Therefore, accelerating or slowing down the theta oscillation should result in a lower or higher working memory capacity, respectively.

**Fig 1:**
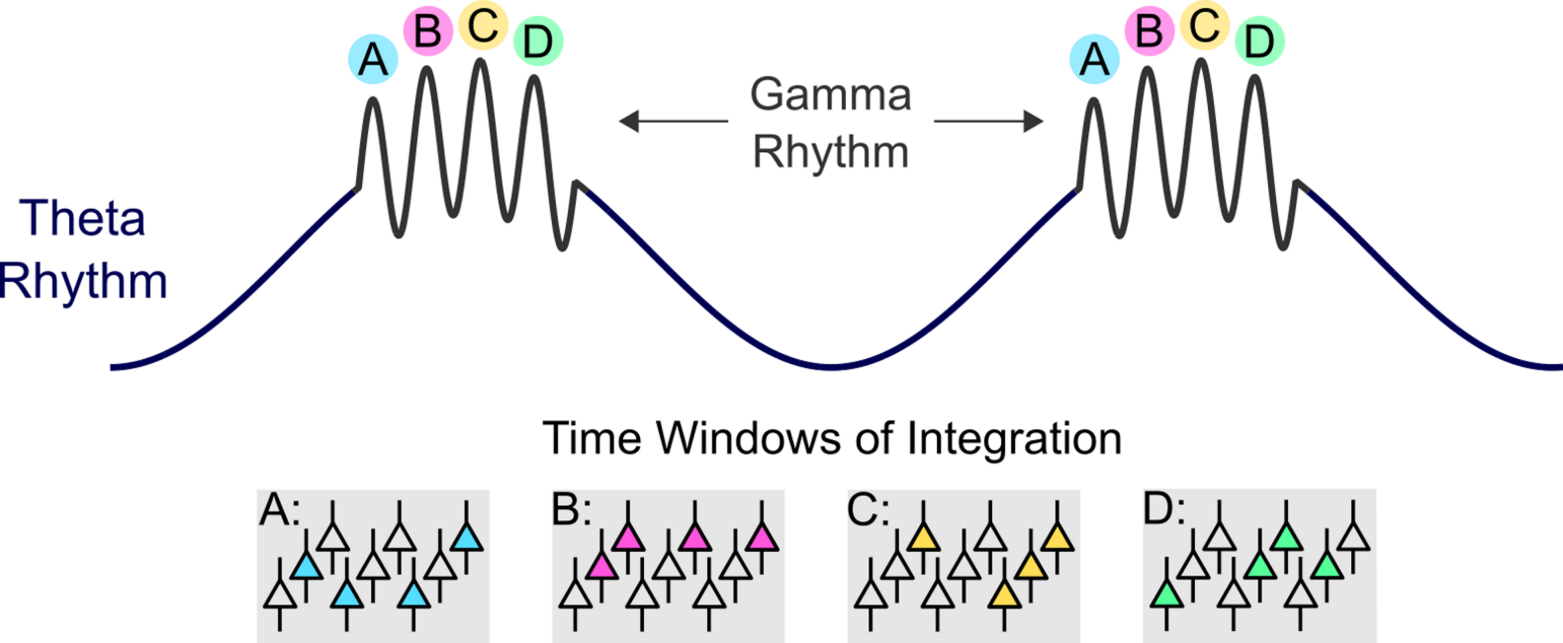
Theta–gamma neural coding scheme. Each gamma cycle represents the activity of local neural assemblies (represented as A, B, C, and D in the figure), each encoding a single item of information. The global theta rhythm organizes multiple gamma cycles by phase–amplitude cross-frequency coupling. According to this concept, the amplitude of the faster rhythm (e.g., gamma) increases during the certain phase of the slower rhythm (e.g., theta).

This intriguing hypothesis begs the use of interventional methods to directly modulate neural activity and study its effect on behavior. Transcranial alternating current stimulation (tACS) is a noninvasive neuromodulation technique in which a weak, oscillating electric current is applied to the neocortex via skin electrodes [7]. The tACS-generated oscillating electric field can modulate and entrain the ongoing network oscillations in a frequency-specific manner [8]. In vitro, in vivo, and human experiments have demonstrated the feasibility of tACS for manipulating the phase, the power, and the rhythm of neural oscillations when appropriate stimulation parameters (i.e., frequency, intensity, and anatomical location) are used [9]. Single- and cross-frequency tACS, together with electrophysiological and neuroimaging methods, offer an unprecedented opportunity to study network oscillations and their possible role in cognition, such as the role of theta–gamma neural code in working memory [10].

In the present issue of *PLOS Biology*, Wolinski and colleagues [2] approached the role of the theta–gamma neural code by stimulating healthy participants with single-frequency tACS in the theta frequency range while the volunteers performed a visuospatial working memory task. In this paradigm, participants memorized 2 arrays of colored squares presented on the left or the right side of the screen. Participants were requested to fixate on the center of the screen while their attention was covertly deployed toward the cued array of colored squares on the left or the right side of the screen. The number of squares in each array varied between 4 and 6. This number is somewhat lower than the classical capacity limit of working memory, described by Miller in his seminal paper to be 7 ± 2 chunks [11]. However, the capacity limit for visual working memory items is somewhat lower, ranging between 3 and 5 items [12]. At the beginning of each trial, the participants were shown an arrow pointing to the left or the right side of the screen, which informed them as to which array they should attend. Two memory arrays were then presented sequentially. During the first presentation, the participants were to memorize the color of the squares. The arrays disappeared and were then shown a second time. Here, the participants had to indicate whether the squares remained the same during the second presentation or if the colors had changed. The authors used the K value measure of visual working memory capacity, which is unbiased to the working memory span. The K value is the difference between the observed hit and false alarm rates, which is multiplied by the number or items to memorize. To test the hypothesis that a lower theta frequency enabled more gamma cycles to be nested than a higher frequency, Wolinski and colleagues applied single-frequency tACS at 4 or 7 Hz. They argued that if tACS entrained ongoing theta oscillation, tACS at 4 Hz would allow the theta cycle to nest-in more item-specific gamma subcycles than tACS at 7 Hz. This is exactly what the authors found; slow theta tACS increased, while the faster theta tACS reduced the visuospatial working memory capacity. Further confirmation for the hypothesis was the observation that the modulation of working memory capacity was spatially specific for presentation in the hemifield contralateral to the stimulated hemisphere. The electrode montage and the resulting electric field also played a role; the effects only occurred with a right supraorbital return electrode but not with a return electrode positioned over the vertex. That is, Wolinski and colleagues only found behavioral effects of tACS when they targeted a relevant node of the working memory network specifically involved in visuospatial working memory tasks, while the control montage had no impact on the behavior.

The study by Wolinski and colleagues raises questions that will lead to new insights into the functional role of neural oscillations. We can generalize the present discussion and envision some future developments. Under their assumption that tACS at the theta frequency does not affect the speed of the gamma rhythm itself, the 4 Hz theta should accommodate almost twice as many gamma cycles as the 7 Hz theta and thus almost double the working memory capacity. However, the authors found only a 7% difference in working memory capacity between the stimulation conditions. The question is why working memory capacity did not increase to a greater extent with 4 Hz tACS. The behavioral impact of the slow and fast theta tACS in Wolinski and colleagues’ study may be explained by the fact that neural assemblies utilize two functionally separable processing periods. In the so-called duty cycle period, neurons send the information to the downstream neurons, but neurons in the duty cycle are less sensitive to incoming information. In the relaxation period, on the other hand, neuronal assemblies do not transfer neural information but are in a sensitive state to be perturbed by the incoming neural activity. This feature, which is reminiscent of the so-called relaxation oscillators, enables synchronization of the activity of a large number of neural assemblies by providing a predictable time window for neural information transfer [1]. However, it also reduces the time window for perturbation by means of external electrical stimulation, which is applied traditionally in a continuous fashion. Given that the ratio of the duty and the relaxation cycles in the theta cycle is unknown, the behavioral results of Wolinski and colleagues will inspire future experimental work to improve the temporal correspondence of the external stimulation relative to the ongoing network oscillations. Future studies may also test the event-related forms of tACS, in which the stimulation is applied for a short period of time, synchronized with a particular working memory event. Because the neural coding scheme during working memory encoding and during the maintenance periods requires two functionally separable modes, event-related tACS is a promising way to test the functional role of different oscillatory modes [13].

In their study, Wolinski and colleagues assumed that externally applied alternating current resonated with a brain rhythm oscillating at approximately the same frequency as the applied tACS, which suggests that the brain rhythm can be forced to accelerate or slow down to achieve a full match (Fig 2). Although the significant and opposite behavioral effects of stimulation with slow versus fast theta tACS support the entrainment hypothesis, the precise electrophysiological mechanisms of the neural entrainment effect require magnetoencephalogram (MEG) and electroencephalogram (EEG) recordings in future experiments. Progress toward this goal has been hindered by the fact that despite the initially promising developments [14], it is still technically challenging to record M/EEG during tACS [15]. As an alternative approach, artifacts may be reduced by using rhythmically applied short pulses, which induce short artifacts, unlike continuous sinusoidal waveforms [16]. Nevertheless, without simultaneous tACS–M/EEG recordings, the exact degree to which the stimulation protocol in Wolinski and colleagues’ study altered the frequency of the natural theta rhythm has yet to be studied in future work.

**Fig 2:**
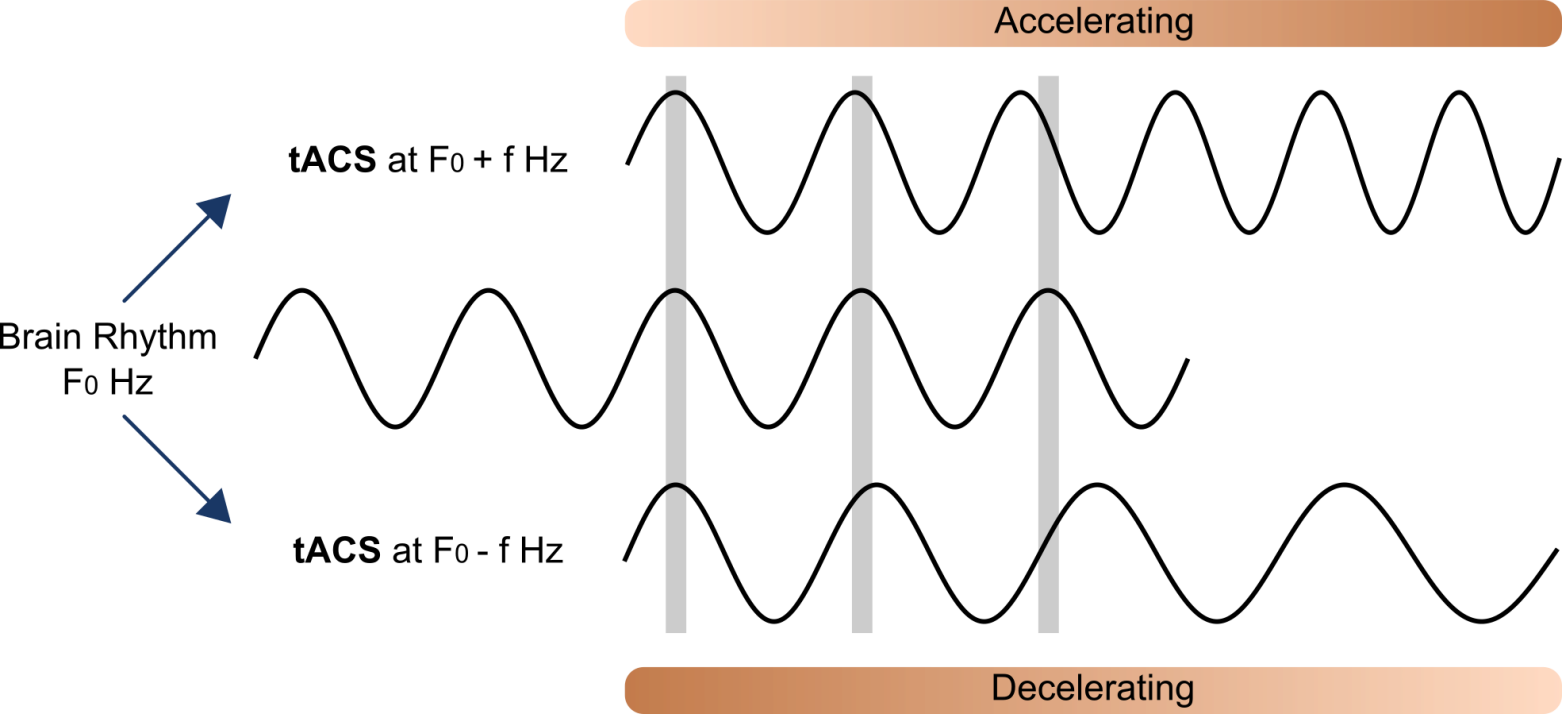
Theoretical framework of frequency entrainment. The basic brain rhythm F_0_ is subjected to an external force (such as tACS) oscillating at a close frequency (F_0_ ± f). If magnitude and timing of the external force are appropriate, the brain rhythm will accelerate or decelerate toward the frequency of the external force. tACS, transcranial alternating current stimulation.

The intriguing behavioral results from Wolinski and colleagues lead us to the question whether it is possible to tailor the stimulation parameters to each individual theta frequency. The authors conclude that biasing the theta rhythm toward a lower frequency (4 Hz) increases the working memory capacity, while biasing toward the faster theta (7 Hz) reduces it. But is a further reduction of the theta frequency even more beneficial for the working memory or other cognitive processes? From a system-level perspective, the various brain rhythms cannot exist too close to each other or to each other’s harmonics. This might, otherwise, create an overlap that could compromise the phase coding role of neural oscillations [11]. Oscillations that are too slow could also impair the ability of the brain to react to the external stimuli in a rapid enough manner. To answer this question, a more “fine-grained” examination of the theta range, which spans from 3 to 8 Hz, will undoubtedly be of interest. Finally, direct manipulations not only with theta but with theta and gamma rhythms would strengthen the arguments in favor of the theta–gamma model [10,17]. According to the Arnold tongue model of oscillatory entrainment, frequency entrainment depends both on the proximity of the stimulation frequency to the individual’s rhythm and on the effective stimulation dose actually reaching the neural tissues [18]. This dose varies due to interindividual differences in head anatomy. Thus, some participants respond to the standard low-intensity stimulation better than the others. An individualized approach promises to improve the effectiveness of tACS.

Based on the computational modeling results performed on a template head model, Wolinski and colleagues targeted a relevant node of the working memory network that is involved in visuospatial working memory. It remains to be seen whether individualized simulations of the spatial distribution of the resulting electric field can further optimize the behavioral effects of the stimulation. Inspired by the behavioral results of Wolinski and colleagues, future studies may also explore whether network stimulation between hemispheres [19] or between lobes [20] can further improve the behavioral impact of the stimulation.

A further perspective for tACS that excites many researchers is its translation into clinical practice. From the biomedical viewpoint, transferring it from bench to bedside in a patient population would require a deeper understanding of tACS-evoked electrophysiological effects and its dose and frequency responses. From the cognitive viewpoint, the main focus of Wolinski and colleagues’ work was directed to the visual working memory capacity. It therefore remains to be seen whether the theta stimulation has other beneficial or detrimental behavioral effects and, if so, whether one can avoid inducing detrimental effects while keeping the beneficial ones with an individualized stimulation.

Modern cognitive neuroscience and computational psychiatry attribute numerous cognitive functions in normal and pathological states to the neural oscillations. In particular, theta rhythm has been associated with goal-directed behavior, including working memory, attention, and decision-making. The theoretical frameworks, such as the theta–gamma neural code, move the research focus from the individual brain rhythms to their hierarchy and interplay. The correlation between the well-known capacity limit of working memory and the number of theta-nested gamma subcycles has attracted the attention of many investigators. By investigating the causal role of theta oscillations in working memory capacity, Wolinski and colleagues are making a thought-provoking contribution that inspires manifold future studies.

## Abbreviations

EEG: electroencephalogram
MEG: magnetoencephalogram
tACS: transcranial alternating current stimulation
